# Zeolite-Containing Mixture Supplementation Ameliorated Dextran Sodium Sulfate-Induced Colitis in Mice by Suppressing the Inflammatory Bowel Disease Pathway and Improving Apoptosis in Colon Mucosa

**DOI:** 10.3390/nu9050467

**Published:** 2017-05-06

**Authors:** Weida Lyu, Huijuan Jia, Chuanzong Deng, Kenji Saito, Seigo Yamada, Hisanori Kato

**Affiliations:** 1 Department of Applied Biological Chemistry, The University of Tokyo, 1-1-1, Yayoi, Bunkyo-ku, Tokyo 113-8657, Japan; weidalvleo@gmail.com; 2 Corporate Sponsored Research Program “Food for Life”, Organization for Interdisciplinary Research Projects, The University of Tokyo, 1-1-1, Yayoi, Bunkyo-ku, Tokyo 113-8657, Japan; kkkj774@yahoo.co.jp; 3 Azuma Chemical Co. Ltd., 3-4-4 Iidabashi, Chiyoda-ku, Tokyo 102-0072, Japan; deng@azumakasei.jp (C.D.); yes-yamada@azumakasei.jp (S.Y.)

**Keywords:** zeolite-containing mixture (Hydryeast^®^), Azumaceramics, DSS, inflammatory bowel disease, apoptosis, colon mucosa

## Abstract

Inflammatory bowel disease (IBD) is induced by multiple environmental factors, and there is still no known treatment capable of curing the disease completely. We propose a zeolite-containing mixture (Hydryeast^®^, HY)—a multi-component nutraceutical of which the main ingredients are Azumaceramics (mixture of zeolite and oyster shell burned under high temperature), citric acid, red rice yeast (monascus) and calcium stearate—as a nutraceutical intervention in IBD to ameliorate dextran sodium sulfate (DSS)-induced colitis. We show the mechanism through integrated omics using transcriptomics and proteomics. C57BL6 mice were given an AIN-93G basal diet or a 0.8% HY containing diet and sterilized tap water for 11 days. Colitis was then induced by 1.5% (*w*/*v*) DSS-containing water for 9 days. HY fed mice showed significantly improved disease activity index and colon length compared to DSS mice. Colonic mucosa microarray analysis plus RT-PCR results indicate HY supplementation may ameliorate inflammation by inhibiting the intestinal inflammatory pathway and suppress apoptosis by curbing the expression of genes like tumor protein 53 and epidermal growth factor receptor and by upregulating epithelial protection-related proteins such as epithelial cell adhesion molecule and tenascin C, thus maintaining mucosal immune homeostasis and epithelial integrity, mirroring the proteome analysis results. HY appears to have a suppressive effect on colitis.

## 1. Introduction

Inflammatory bowel diseases (IBD), which include Crohn’s disease (CD), ulcerative colitis (UC), and indeterminate colitis (IC), are characterized by chronic, remittent, and inflammatory-mediated disorders of the gastrointestinal tract [1]. The precise etiology of IBD is not fully understood, but it has gradually become clear that IBD involves the interactions of multiple environmental factors like foods and smoking [2], diverse properties of predisposing genetic factors, and immune system response [3] such as innate and adaptive immune abnormal regulation which results in responses to the intestinal microbiota in genetically susceptible hosts [4]. There is still no effective treatment that heals IBD completely, and affected patients continue to suffer from IBD symptoms along with a tendency to relapse.

Hydryeast^®^ (HY, Azuma Chemical Co. Ltd., Tokyo, Japan) is a zeolite-containing mixture that is a multi-component nutraceutical with the following main ingredients: Azumaceramics (47.6%), citric acid (33.3%), red rice yeast (monascus) (16.7%), and calcium stearate (2.4%). The major component is the Azumaceramics, which is a mixture of zeolite and oyster shell burned under high temperature. Zeolite, a mineral with a microporous structure, is used mainly as a cation exchanger by a chemical/physical process in several industries and in the field of medicine [5], and zeolite is also added to the fodder of livestock and poultry, as it can improve the absorptivity and bioavailability of some minerals and vitamins [6,7]. Citric acid is a weak organic tribasic acid, the chemical formula of which is C_6_H_8_O_7_, and its main applications are as a food additive and in the fields of chemistry and textiles. Red rice yeast (monascus), produced from the fermentation of steamed rice using the fungus *Monascus purpureus*, is widely utilized in food coloring and preserving and wine making; moreover, in East Asia, red rice yeast has been used for centuries to promote food digestion and blood circulation [8]. Calcium stearate is an anticaking agent used in a wide variety of foods including salt, spices, confections, snack products, and dry mixes. To the best of our knowledge, little or no research has been conducted regarding the application of the components described above as a treatment for IBD.

In this study, we used a nutrigenomics method to gain a better understanding of HY as a dietary supplement for the improvement of colitis in a mouse model of dextran sodium sulfate (DSS)-induced colitis through transcriptome, proteome, and biochemical analyses using colon mucosa tissue.

## 2. Materials and Methods

### 2.1. Animals and Dietary Treatment

Seven-week-old male C57BL6 mice obtained from Charles River Japan (Tokyo, Japan) were housed in individual animal cages at controlled temperature (23 ± 2 °C), relative humidity (50–60%), and lighting condition (12-h light/dark cycle) throughout the entire experiment. After 3 days of acclimatization, the mice were assigned to four groups with equal mean body weights, with each group comprised of six to eight mice: (1) the HY8 group, which received the 0.8% HY powder added AIN-93G basal diet and sterilized tap water during the entire experiment; (2) the CON group, which received the AIN-93G basal diet and sterilized tap water for the course of the entire experiment; (3) the DSS group, which received the AIN-93G (American Institute of Nutrition in 1993) basal diet and sterilized tap water for 11 days, after which colitis was induced by 1.5% (*w*/*v*) DSS (DSS molecular weight, 40 kDa; MP Biomedicals, Irvine, CA, USA)-containing sterilized tap water for 9 days; (4) the DHY8 group, which received the 0.8% HY powder added AIN-93G basal diet during the entire experiment and sterilized tap water for 11 days, after which colitis was induced by 1.5% DSS-containing sterilized tap water for 9 days. The 0.8% HY added diet was adjusted with cornstarch to maintain the caloric balance, as shown in Table 1. The choice of 0.8% HY in this experiment was based on our preliminary dose-dependence experiment [9]. This research was approved by the Animal Care and Use Committee of the University of Tokyo (Approval No. P13-739).

### 2.2. Evaluation of the Disease Activity Index

The Disease Activity Index (DAI) was the average of three scores (body weight loss, fecal blood, and stool consistency) after the DSS was fed to mice. These scores were determined as follows: body weight loss ratio (0: <1%, 1: 1–5%, 2: 5–10%, 3: 10–15%, 4: >15%), fecal blood (0: no fecal blood, 2: ++, 4: +++) and stool consistency (0: normal, 2: soft, 4: diarrhea) [10].

### 2.3. Blood Collection and Tissue Harvesting

Upon termination of the experiment, all of the mice were deeply anesthetized with pentobarbital sodium prior to euthanasia by bleeding from the carotid artery. The blood obtained was centrifuged at 1000× *g* for 15 min at 4 °C to obtain the plasma. The colon length was measured between the ileo-cecal junction and the proximal rectum, and this measurement was also used as one of the criteria for the extent of colitis. The tissues of liver, excised colon, mesenteric adipose, and retroperitoneal adipose were snap-frozen in liquid nitrogen and stored at −80 °C until further analysis.

### 2.4. Biochemical Assays

Colonic myeloperoxidase (MPO) activity was measured by the colorimetrical method using the MPO Activity Colorimetric Assay Kit (BioVision, Palo Alto, CA, USA) according to the manufacturer’s instructions.

### 2.5. Colon Histology

Each colon slice was embedded in OCT (Optimal Cutting Temperature) compound (Sakura Finetek, Torrance, CA, USA) and then snap-frozen in liquid nitrogen. Each 5-μm-thick slice of tissue was sectioned and stained by hematoxylin and eosin (H&E), then scanned by light microscopy (Olympus BX51 microscope, Olympus Optical, Tokyo, Japan).

### 2.6. Total RNA Extraction and Quality Assessment

Total RNA was extracted from the colon mucosa using the total RNA Isolation Kit, NucleoSpin^®^ RNAΠ (Macherey-Nagel, Düren, Germany) following the manufacturer’s instructions. The concentration and purity of RNA were measured with a NanoDrop ND-1000 spectrophotometer (NanoDrop Technologies, Wilmington, DE, USA).

### 2.7. Transcriptome Analysis

#### 2.7.1. DNA Microarray Preparation

The colon mucosa RNA samples from individual mice in each group were pooled (*n* = 6–8), and a microarray analysis was then conducted using Affymetrix Mouse Genome 430 2.0 Array Genechips (Affymetrix, Santa Clara, CA, USA), which contains over 40,000 gene probe sets for genome-wide expression profiling.

#### 2.7.2. Mapping and Functional Analysis

We compared gene expression ratios between the DSS and CON groups and between the DHY8 and DSS groups after the images were scanned by Microarray Suite ver. 5.0 software (Affymetrix, Santa Clara, CA, USA). An increase in an expression ratio between treatments that was >1.5-fold was regarded as significant expression.

### 2.8. Reverse Transcription-Polymerase Chain Reaction

To verify the expression of differentially expressed genes, we carried out a reverse transcription-polymerase chain reaction (RT-PCR), the primer sequences of which are shown in Table S1. The expression levels of respective genes were normalized against the expression of 60S acidic ribosomal protein p1 (*Rplp1*) in colon mucosa.

### 2.9. Protein Preparation, iTRAQ Labeling, and NanoLC-MS/MS Analysis for Proteome Analysis

Lysis buffer was used to extract protein from colon mucosa, which was then separated by centrifugation at 12,000× *g* for 30 min at 4 °C. Protein concentrations were determined by a Bradford assay. According to the manual of the 4-plex iTRAQ labeling kit (AB Sciex, Framingham, MA, USA), proteins (100 μg) were pooled for cysteine blocking and digested, then labeled with isobaric tags as follows for the further analysis by nanoscale liquid chromatography coupled to tandem mass spectrometry (NanoLC-MS/MS; AB Sciex, Tokyo, Japan): CON, 115 tag; DSS, 114 tag; DHY8, 116 tag.

### 2.10. Statistical Analysis

The data are presented as the mean value ± standard error (SE) and were analyzed by a two-way analysis of variance (ANOVA). Significant differences were evaluated with Tukey’s test at the level of *p* < 0.05.

## 3. Results

### 3.1. General Characteristics

**Figure 1:**
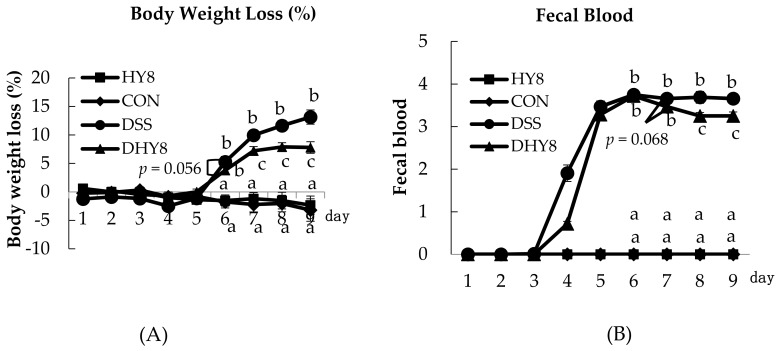

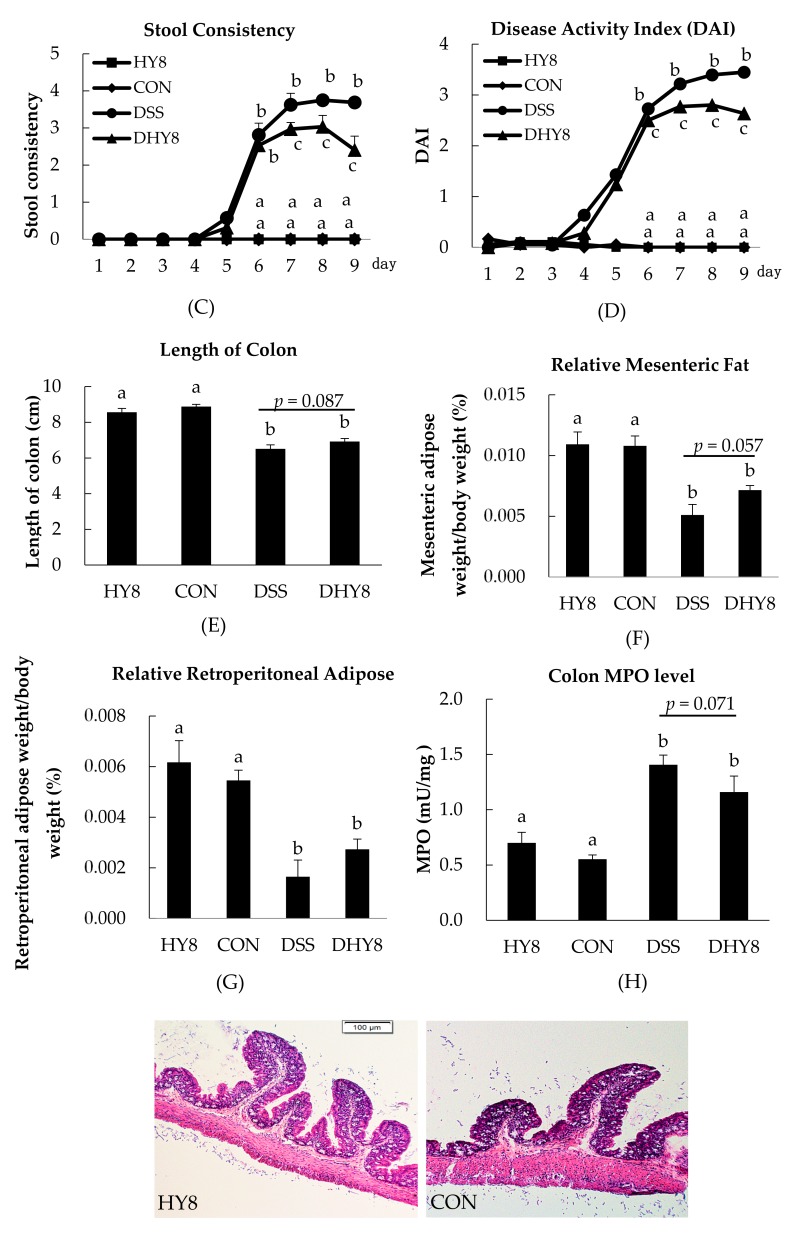

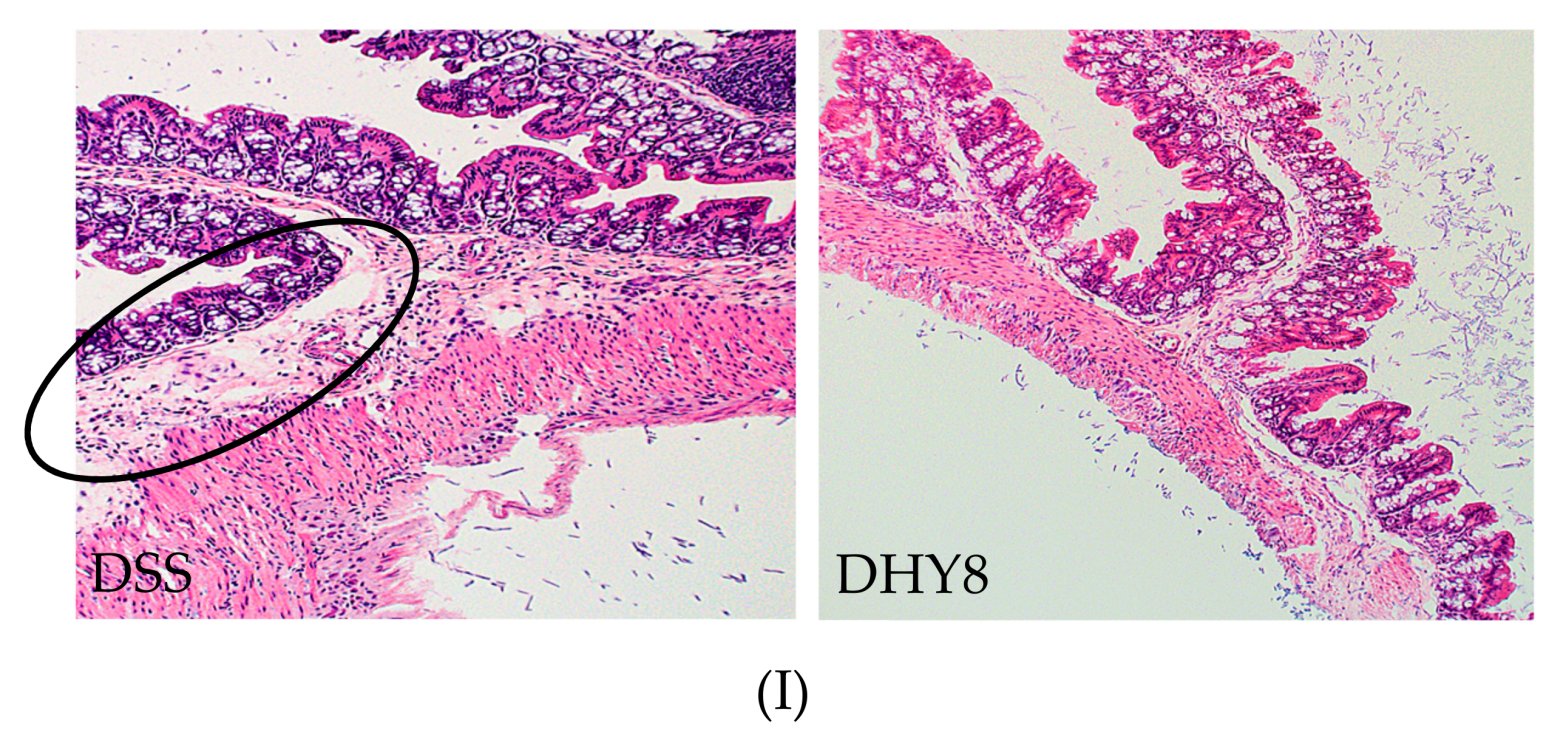
General characters of each groups. (**A**) Body weight loss; (**B**) Fecal blood; (**C**) Stool consistency; (**D**) Disease Activity Index (DAI); (**E**) Length of colon; (**F**) Relative mesenteric fat; (**G**) Relative retroperitoneal adipose; (**H**) Colon MPO level; (**I**) Hematoxylin and eosin (H&E) staining of colon. All values are mean ± SE (*n* = 6–8) by two-way ANOVA.

Some general characteristics were measured to investigate the effect of HY supplementation on colitis. No significant differences were observed in food intake between the DHY8 and DSS groups, but there was a significant difference in food intake between the CON and DSS groups (HY8: 76.7 ± 2.6; CON: 82.8 ± 3.5; DSS: 73.1 ± 1.7; DHY8: 76.4 ± 3.3 g). There was a significant difference in water intake between the HY8 and DSS groups (HY8: 84.8 ± 2.3; DSS: 70.3 ± 2.1; CON: 73.7 ± 1.9; DHY8: 72.2 ± 1.4 mL). Severe clinical phenomena occurred following the DSS treatment, including body weight loss and altered fecal blood and stool consistency, which led to deteriorated DAI values compared to the CON group (the clinical scores are shown in Table S4). HY supplementation ameliorated the body weight loss and ameliorated the pathological condition of colitis, which was also indicated by the DAI values on days 6–9 (Figure 1A–D). The DSS-induced decrease in the colon length and mesenteric fat weight as well as the increase of colon MPO activity tended to be improved by HY supplementation, while there was no change in the weight of retroperitoneal adipose tissue (Figure 1E–H).

### 3.2. Colon Histology

Colon sections were made to directly confirm the effect of HY supplementation on colon tissue, especially on colon mucosa. DSS induced a structural disorder of mucosa epithelium cells, which may lead to an increased infiltration of inflammatory cells into the mucosa and submucosa. HY supplementation clearly ameliorated this disorder with a reduced infiltration of inflammation cells (Figure 1I).

### 3.3. Colonic Microarray Analysis

We also carried out the microarray analysis to identify the effect of HY supplementation on colitis from the gene level. A total of 5238 genes were differentially expressed, of which 2300 were significantly upregulated in the DHY8 mice compared to the DSS mice and downregulated in the DSS mice compared to the CON mice, whereas 2938 genes were notably downregulated in the DHY8 group compared to the DSS group and upregulated in the DSS group compared to the CON group. The list of relative gene changes is appended in Table S2.

**Figure 2:**
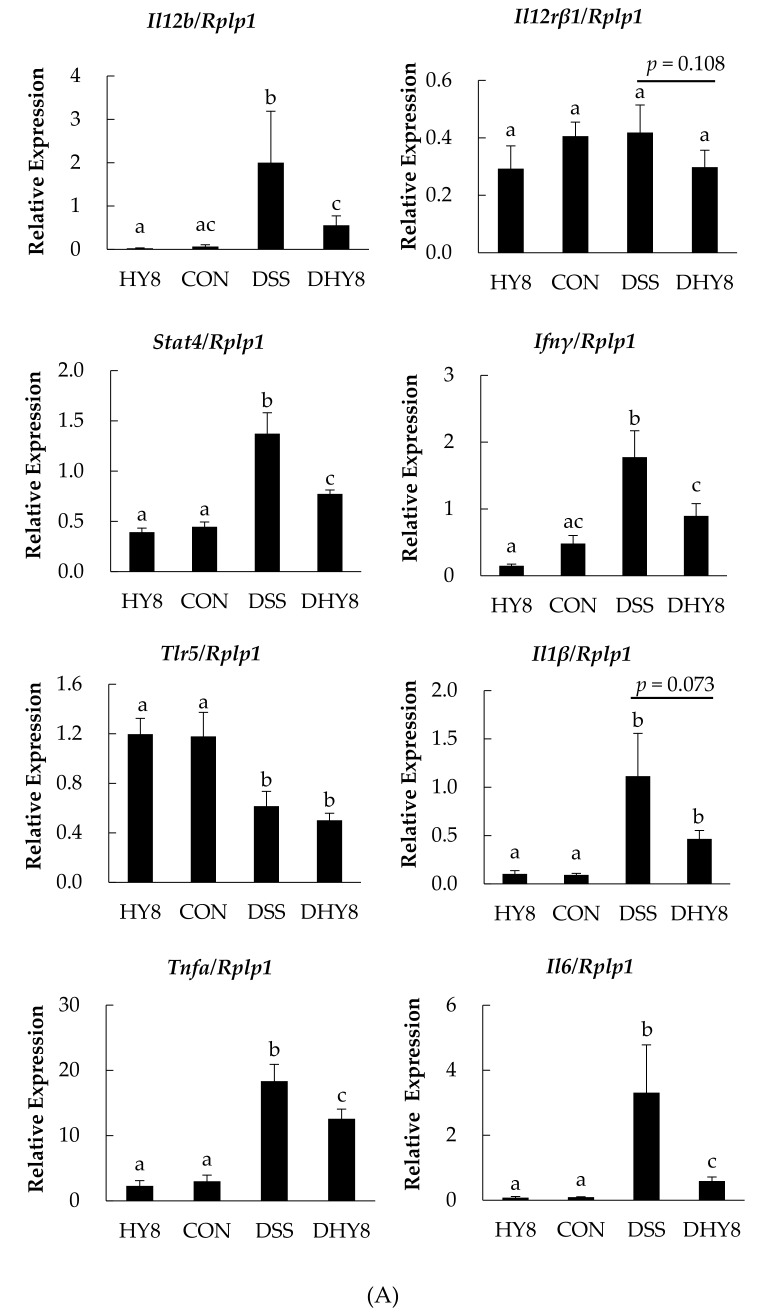

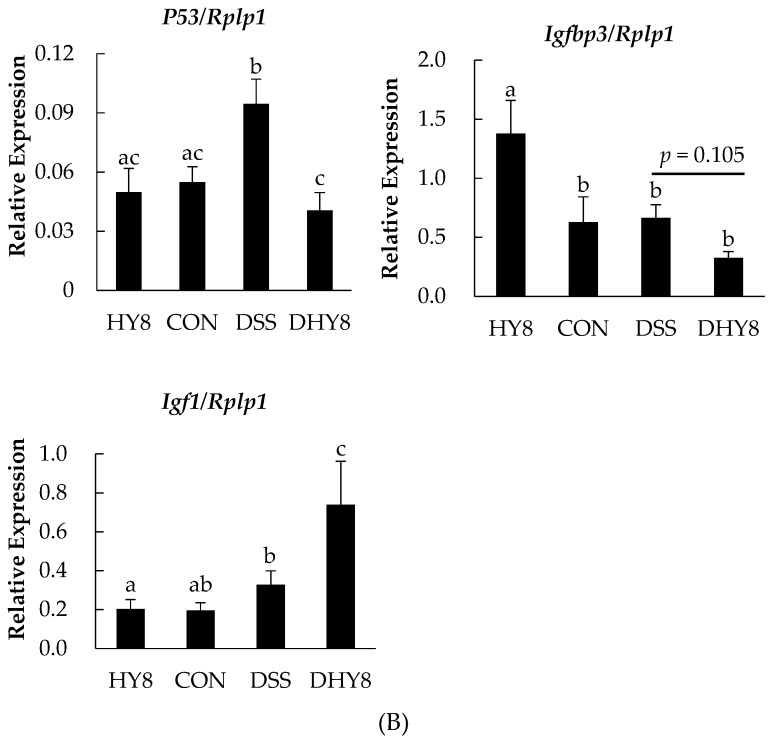
(**A**) Colonic mucosa mRNA expression of genes related to the intestinal inflammatory pathway; (**B**) Apoptosis-related *P53* pathway. The relative mRNA expressions of *Il12b*, *Il12rβ1*, *Ifnγ*, *Stat4*, *Tlr5*, *Il1β*, *Tnfa*, *Il6*, *P53*, *Igfbp3*, and *Igf1* were measured by RT-PCR and normalized to *Rplp1*. All values are mean ± SE (*n* = 6–8) by two-way ANOVA.

HY supplementation ameliorated the inflammation level in colon mucosa via suppressing the expression of genes related to the intestinal inflammatory pathway, including interleukin 12b (*Il12b*), signal transducer and activator of transcription 4 (*Stat4*), interferon gamma (*Ifnγ*), tumor necrosis factor-alpha (*Tnfα*), interleukin 6 (*Il6*); the expression of interleukin 12 receptor subunit beta 1 (*Il12rβ1*) and interleukin 1 beta (*Il1β*) showed a decreasing tendency (*p* = 0.108, *p* = 0.073) (Figure 2A). HY supplementation also downregulated the expression of the following genes: apoptosis-related tumor protein 53 (*P53*) (Figure 2B); members of the chemokine ligand family (*Cxcl*): *Cxcl1*, *Cxcl2*, *Cxcl3*, *Cxcl10*, *Cxcl12*, and *Cxcl17*, chemokine (C-C motif) ligand (*Ccl*): *Ccl4* and *Ccl24*; and an epithelial maintenance-related gene, epidermal growth factor receptor (*Egfr*). Compared to the DSS group, trefoil family factor 2 (*Tff2*) and insulin-like growth factor 1 (*Igf1*) were upregulated in the DHY8 group (Figure 2B).

### 3.4. Comparative Proteomic Analysis by iTRAQ

We then validated the effect of HY supplementation on colitis from the protein level by iTRAQ. More than 5000 proteins were identified by the comparative proteomic analysis by iTRAQ. We regarded the proteins that exhibited >1.5-fold changes as altered proteins, and 668 proteins were changed to this degree, including 332 proteins that were upregulated in the DHY8 mice compared to the DSS mice but downregulated in the DSS mice compared to the CON mice. There were 336 proteins that were downregulated in the DHY8 group compared to the DSS group but upregulated in the DSS group compared to the CON group (Table S3).

The 668 altered proteins were put into Ingenuity Pathway Analysis (IPA) [11] to further investigate the relative protein alteration associated with colitis. Supplementation with HY improved the inflammation, apoptosis, intestinal motility, and epithelia condition by upregulating the following proteins: a protein involved in cancer, periostin (Postn); the anti-inflammation-related proteins adenylate cyclase activating polypeptide (Perp1) and galectin 2 (Leg2); the anti-apoptosis-related proteins known as BCL2-associated athanogene 3 (Bag3), RNA-binding motif protein 3 (Rbm3), and ferritin heavy chain (Fhc); an intestinal motility-related protein, phosphatase 1 regulatory inhibitor subunit 14A (Pp14a); and some intestinal mucosal protection and epithelia homeostasis-related proteins, i.e., epithelial cell adhesion molecule (Epcam), tenascin C (Tena), and numb homolog (Numb) (Table 2).

Inflammation and apoptosis induced by DSS could also be proved to be improved by the decreased amounts of the following proteins: inflammatory degree-related proteins, i.e., haptoglobin (Hpt), complement component 3 (Co3), myeloperoxidase (Perm), Annexin A2 (Anxa2), and heat shock protein family A member 4 (Hsp74); proteins involved in apoptosis, i.e., keratin 20 (K1c20), apyrimidinic endodeoxyribonuclease 1 (Apex1), caspase-7 (Casp7), and caspase-3 (Casp3); and a protein of biomarker for colorectal carcinoma, heterogeneous nuclear ribonucleoprotein U (Hnrpu) (Table 2).

## 4. Discussion

**Figure 3:**
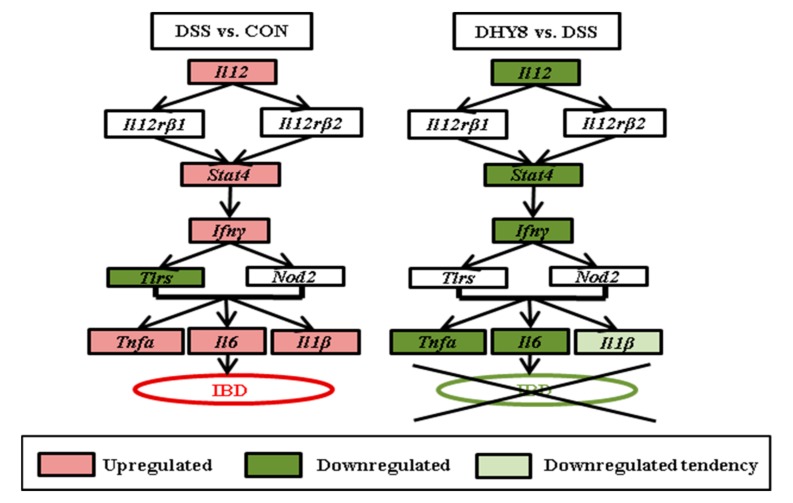
Schematic representation of the intestinal inflammatory pathway expression altered by Hydryeast supplementation (DHY8) in dextran sodium sulfate (DSS)-induced colitis.

The microarray analysis revealed that HY supplementation ameliorated the inflammatory status in the mice by suppressing the expression of genes involved in the intestinal inflammatory pathway: *Il12*^pos^ *Il12rβ1*^pos^ *Stat4*^pos^ *Ifnγ*^pos^ *Tlr5*^pos^ *Tnfα*/*Il6*/*Il1β*. Figure 3 is a schematic representation of the intestinal inflammatory pathway expression altered by HY supplementation in DSS-induced colitis. In this pathway, Stat4 tyrosine phosphorylation is induced by IL-12; this is required for the T-cell-independent induction of the cytokine IFN-*γ* [12], and then in response to the TLR stimulation, both lymphoid and non-lymphoid cells can produce the pro-inflammatory cytokine IL6 [13], which together with TNFα and IL1β are considered to be the major pro-inflammatory cytokines. The downregulation of the intestinal inflammatory pathway led mainly to decreased levels of the pro-inflammatory cytokines induced by DSS, which by this point indicates the mitigated inflammatory degree by HY treatment.

The above-mentioned reduction of pro-inflammatory cytokine levels may also be reflected in the altered expression of the following three marker proteins. (1) The upregulation of Pp14a (Cpi-17), which is an inhibitor of smooth muscle myosin phosphatase and would be suppressed by the upregulation of IL1β or TNFα induced by chronic inflammatory bowel diseases and then lead to dysfunctional motility [14,15]; (2) The downregulation of Hpt, an acute-phase α-sialoglycoprotein with hemoglobin-binding capacity which could be induced by pro-inflammatory cytokines such as IL6, IL1β, and TNFα [16]; (3) The reduction of Hsp74, which could be induced by inflammation and might then result in the inhibition of apoptosis of inflammatory cells and enhance the immune response by increasing *Bcl-2* and *Il-12* expression [17].

The alteration of the following three proteins also indicated the amelioration of inflammation by HY supplementation. (1) The upregulation of Perp1, which plays a key role in anti-inflammation by controlling the balance of Th1/Th2 and reducing the secretion of pro-inflammatory cytokines such as IL6, IL1β, and TNFα. Perp1 is also associated with the maintenance of intestinal epithelial integrity, and a deficiency of Perp1 would thus result in damage to epithelial cells of intestine [18]; (2) The downregulation of Co3, which plays a key role in the activation of the complement system. The reduction of Co3 expression would lessen the number of infiltrating neutrophils in the lesions (which are the main producer of IL1β) and thus function as tumor suppression [19]; (3) The diminution of Anxa2 could downregulate another pro-inflammatory cytokine, TNFα, by the prevention of TNFα shedding in IBD [20]. Taking the findings regarding the significantly changed genes and relative proteins induced by HY supplementation together, we propose that HY supplementation might ameliorate DSS-induced inflammation by mainly suppressing the intestinal inflammatory pathway as well as relative genes and proteins, and then suppressing the expression of the pro-inflammatory cytokine genes *Il6*, *Il1β*, and *Tnfα*.

In addition to the anti-inflammation effect of HY treatment, we speculate that HY supplementation may alleviate DSS-induced colitis by ameliorating apoptosis, regulating the cell cycle, and preserving epithelial integrity, thus maintaining the barrier function of colon mucosa. In relation to the *P53* gene, which was shown to be downregulated in microarray analysis, we investigated the expression of the pathway *P53*^pos^ *Igfbp3*^neg^ *Igf1*, the positive expression of which would lead to apoptosis. However, the expression of the gene *Igfbp3* was not significantly different between the DSS and DHY8 groups. The upregulation of the matricellular protein Postn revealed by the proteome analysis also confirmed our hypothesis, as its overexpression may result in the downregulation of *P53*, the overexpression of which would lead to apoptosis [21]. We thus suspect that the addition of HY upregulated Postn and then inhibited the expression of *P53*, by which the apoptosis was suppressed and the cell growth and the cell cycle were improved. Moreover, the downregulation of *P53* could also lead to a decrease in the production of DNA-damaging molecules, which could result in inflammation and then apoptosis and cell loss [22]. The reduced expression of *P53* might not only indicate the anti-apoptosis function of HY but might also ameliorate the inflammatory status induced by DSS. From the results of the RT-PCR, we found that between the HY8 and CON groups there are no significant differences in the gene expressions of *Il12b*, *Il12rβ1*, *Stat4*, *Ifnγ*, *Tnf-α*, *Il6*, *Il-1β*, *P53*, and *Igf1* except the result of *Igfbp3*. These findings indicate that HY might have no direct side effect on mice as compared to the CON group. However, future experiments are needed to confirm this.

In addition, the ameliorated apoptosis may be echoed by the upregulation of another three related proteins: Bag3, which is capable of suppressing apoptosis by Bcl-2 in vitro [23] and maintaining cell survival by restraining cell death and regulating gastrointestinal functions [24]; Rbm3, the lack of which could lead to mitotic catastrophe due to the absence of important proteins required for cell growth [25,26]; and Fhc, which may function as an essential mediator to antagonize apoptosis by inhibiting the activation of mitogen-activated protein kinase 8 (Mapk8) through iron sequestration (which is related to reactive oxygen species accumulation) and thereby suppressing the apoptosis induced by TNFα [27].

In light of the results of our microarray analysis showing downregulation of *Mapk8* (DSS vs. CON: 1.49, DHY8 vs. DSS: 0.07) and the PCR result for *Tnfα*, we speculated that the supplementation of HY might also suppress apoptosis by promoting the pathway *Fhc*^neg^*Mapk8*^pos^*Tnfα*. In addition to the three upregulated proteins, the downregulation of another three apoptosis-related proteins may further confirm our speculation: Apex1, which could lead to the gathering of damaged DNA by affecting the DNA repair process via a reduction of the ability to renovate oxidative damage, thus promoting the rates of apoptosis and increasing the risk of UC [28,29]; Casp3, the overexpression of which is also reported to lead to increased apoptosis and a decline in proliferation capacity [30]; and K1c20, a marker of small intestinal goblet cells, which can be induced by apoptosis and tissue injury [31].

The expression of another two genes could be of great importance in the maintenance of epithelial cells and barrier function both directly and indirectly. The activation of *Egfr*—which is upregulated in AOM (azoxymethane)/DSS-induced colitis and also induced by colitis—plays a role in the amelioration of damaged epithelial cells and in the maintenance of the epithelial barrier function in UC [32]. The downregulation of *Egfr* by HY supplementation indicates the improvement of colitic status. *Tff2*, which is secreted and expressed by gastrointestinal epithelium and plays a vital role in the maintenance of epithelial integrity and mucosal surfaces [33], was upregulated by HY supplementation. Moreover, Tff2 is essential in the immune response in inflammatory conditions by controlling the expression of pro-inflammatory cytokines such as IL6 and IL1β [34].

In the present study, the changes of *Egfr* and *Tff2* expression brought about by HY treatment indicated the amelioration of epithelial cells damaged by DSS, which might cause a lesion to the intestinal barrier and promote the permeability of barrier cells. The above-described findings might be supported by the colon histology results showing that the status of the goblet cells damaged by DSS was alleviated in the DHY8 mice compared to the DSS mice. In addition, TFF2 deficiency has been reported to lead to a loss of body weight [33], and the upregulation of TFF2 induced by HY supplementation seems consistent with improved body weight loss.

Based on the results of our proteome analysis, we speculate that HY treatment might have contributed to epithelial protection by upregulating the following proteins. Epcam, which plays a role in the shaping of the intestinal normal architectural structure and managing Ca^2+^-independent homotypic cell–cell adhesion, is downregulated in IBD patients, resulting in the loss of epithelial cell contact, increased permeability, and then the penetration of the bacterial products [35,36]. Tena, an extracellular matrix protein, may contribute to intestinal mucosal protection by promoting cell migration and remodeling, accelerating the recovery process in wound areas [37]; moreover, an anti-inflammatory effect of Tena was reported in a study in which the upregulation was shown to inhibit T-cell activation [38]. Numb, a membrane-bound protein that is predominantly expressed in intestinal mucosa and might determine cell fate, could lead to an improvement of intestinal epithelial barrier function via a modulation of the paracellular permeability by affecting the apical junctional complex assembly and myosin light chain phosphorylation; a Numb deficiency might result in barrier dysfunction [39].

In the present study, the upregulations of Epcam, Tena, and Numb indicated that the mucosal barrier repair or protection was enhanced and the dysfunction of epithelial adhesion and tight junctions induced by DSS were ameliorated. The above-described results are consistent with our observations of (1) the repaired epithelial cells from the colon histology analysis and (2) improved clinical symptoms, as the status of fecal blood and stool consistency was better in the DHY8 mice compared to the DSS mice.

Regarding the ingredients of HY, the RT-PCR results shown in Figures S1 and S2 suggested that zeolite and citric acid functioned independently, in that zeolite played a role in ameliorating apoptosis by decreasing the expression of *P53* and upregulating the expression of *Igf1*, whereas citric acid significantly suppressed inflammation by inhibiting the intestinal inflammatory pathway. From this viewpoint, we believe that the functions of HY may be the result of interactions of the various HY ingredients.

## 5. Conclusions

Based on our observation of the downregulation of relative pro-inflammation cytokines and the increased expression of the anti-inflammatory factors through our colon transcriptome and proteome analyses, we propose that HY suppresses the expression of the intestinal inflammatory pathway, thus diminishing the degree of inflammation in DSS-induced colitis. HY may suppress apoptosis and maintain mucosal immune homeostasis by inhibiting relative genes such as *P53*; other epithelial maintenance-related genes and proteins were also greatly altered, which indicates that the cell-cycle disorder caused by DSS was improved to some extent. Collectively, the integrated omics analysis results indicate that the effects of HY on DSS-induced colitis are based mainly on ameliorating the inflammation status, improving the degree of apoptosis, and maintaining the epithelial integrity and barrier function of colon mucosa.

## Figures and Tables

**Table 1:** Composition of the diets. HY, Hydryeast^®^.

Ingredients	Control Diet (AIN-93G)	Zeolite-Containing Mixture 0.8%
Cornstarch	39.75%	38.95%
Casein (85.7% protein)	20%	20%
Detrinized cornstarch	13.2%	13.2%
Sucrose	10.25%	10.25%
Soybean oil (no additives)	7%	7%
Fiber	5%	5%
Mineral mix (AIN 93G-MX)	3.5%	3.5%
Vitamin mix (AIN 93G-MX)	1%	1%
L-Cystine	0.3%	0.3%
Zeolite-containing mixture (HY)	0	0.8%

**Table 2:** Differentially expressed proteins in colonic mucosa.

Accession No.	Protein Symbol	Protein Name	Gene Symbol	Fold Change	Fold Change
				DSS vs. CON	DHY8 vs. DSS
Q62009-5	Postn	Isoform 5 of Periostin	*Postn*	0.31	2.36
Q9D8I1	Perp1	Plasma cell-induced resident endoplasmic reticulum protein	*Pacap*	0.13	5.01
Q9CQW5	Leg2	Galectin-2	*Lgals2*	0.62	3.02
Q9JLV1	Bag3	BAG family molecular chaperone regulator 3	*Bag3*	0.67	2.31
O89086	Rbm3	Putative RNA-binding protein 3	*Rbm3*	0.43	4.25
Q8VHX6-2	Fhc	Ferritin heavy chain	*Fhc*	0.37	2.99
Q91VC7	Pp14a	Protein phosphatase 1 regulatory subunit 14A	*Ppp1r14a*	0.33	6.43
Q99JW5	Epcam	Epithelial cell adhesion molecule	*Epcam*	0.32	3.19
Q80YX1-5	Tena	Isoform 5 of Tenascin	*Tnc*	0.65	2.31
Q9QZS3-3	Numb	Isoform 71 kDa of Protein numb homolog	*Numb*	0.44	4.17
Q61646	Hpt	Haptoglobin	*Hp*	36.31	0.32
P01027	Co3	Complement C3	*C3*	6.55	0.34
P11247	Perm	Myeloperoxidase	*Mpo*	5.50	0.55
Q61316	Hsp74	Heat shock 70 kDa protein 4	*Hspa4*	1.89	0.10
Q9D312	K1c20	Keratin, type I cytoskeletal 20	*Krt20*	1.98	0.50
P28352	Apex1	DNA-(apurinic or apyrimidinic site) lyase	*Apex1*	1.92	0.07
P70677	Casp3	Caspase-3	*Casp3*	1.61	0.14
P97864	Casp7	Caspase-7	*Casp7*	6.61	0.42
Q8VEK3	Hnrpu	Heterogeneous nuclear ribonucleoprotein U	*Hnrnpu*	1.92	0.34

CON (control group): AIN-93G basal diet and sterilized tap water; DSS (DSS (dextran sodium sulfate) group): AIN-93G basal diet and sterilized tap water for 11 days, after which colitis was induced by 1.5% (w/v) DSS -containing sterilized tap water for 9 days; DHY8 (DHY8 group), 0.8% HY powder added AIN-93G basal diet during the entire experiment and sterilized tap water for 11 days, after which colitis was induced by 1.5% DSS-containing sterilized tap water for 9 days.

## Supplementary Materials

The following are available online at www.mdpi.com/2072-6643/9/5/467/s1.
